# Pain has limited predictive value for fractures following falls in older people: insight from a retrospective study

**DOI:** 10.1186/s12873-026-01644-2

**Published:** 2026-06-16

**Authors:** Vera Pedersen, Alina Lampart, Rebecca Wania, Isabelle Arnold, Nina Maeder, Sandra Niedermeier, Robert Stahl, Christoph Trumm, Christian Kammerlander, Wolfgang Boecker, Christian H. Nickel, Roland Bingisser, Matthias Klein

**Affiliations:** 1 Department of Orthopedics and Trauma Surgery, Musculoskeletal University Center Munich (MUM), LMU University Hospital, LMU Munich, Marchioninistr. 15, 81377 Munich, Germany; 2 Department of Pulmonology, University Hospital Zurich, Raemistr. 100, Zurich, 8091 Switzerland; 3 Department of Orthopedics and Trauma Surgery, Klinikum Dritter Orden, Menzinger Str. 44, 80638 Munich, Germany; 4 Department of Internal Medicine, University Hospital Basel, Petersgraben 2, Basel, 4031 Switzerland; 5 Department of Gynaekology, Hospital Rheinfelden, Riburgerstr. 12, Rheinfelden, 4310 Switzerland; 6 Department of Anaesthesiology and Intensive Care Medicine, ISAR Klinikum, Sonnenstr. 24–26, 80331 Munich, Germany; 7 Institute for Diagnostic and Interventional Neuroradiology, LMU University Hospital, LMU Munich, Marchioninistr. 15, 81377 Munich, Germany; 8 Radiologie Augsburg Friedberg BAG, Froelichstr. 7, 86150 Augsburg, Germany; 9 Trauma Hospital Styria, Goestinger Straße 24, Graz, 8020 Austria; 10 Department of Emergency Medicine, University Hospital Basel, Petersgraben 2, Basel, 4031 Switzerland; 11 Department of Neurology and Emergency Department, LMU University Hospital, LMU Munich, Marchioninistr. 15, 81377 Munich, Germany

**Keywords:** Low-energy fall, Older adult, Complaints, Fracture, Emergency imaging, Test performance

## Abstract

**Background:**

In the emergency department (ED), decisions regarding radiological imaging for older patients who have experienced low-energy falls (LEF) are frequently complicated by inconsistencies between the reported mechanism of injury, the patients’ symptoms, and the findings on physical examination. Our study aimed to investigate the coherence between physician-documented complaints, the implementation of radiological imaging and the diagnosis of fractures of the vertebral column, rib cage and pelvic ring.

**Methods:**

This is a secondary analysis of a retrospective consecutive sample of 2882 patients presenting with LEF to two urban Level I trauma centers between 1 January 2016 and 31 December 2016, who underwent radiological imaging to diagnose their fractures. Physician-documented complaints were abstracted from electronic health records.

**Results:**

A total of 2882 patients were included. Mild signs of concussion (e.g. headache) (22.0%), extremity pain (18.0%) and pain of the pelvic ring (14.2%) occurred most frequently. Physician-documented complaints were significantly associated with radiological imaging in the respective regions. True prevalence of fractures ranged from 0.14 (95%CI: 0.09, 0.20) in the cervical spine to 0.36 (95%CI: 0.30, 0.41) in the pelvic ring. The sensitivity of documented pain for fractures ranged from 0.78 (95%CI: 0.56, 0.93) in the thoracic spine to 0.98 in the rib cage (95%CI: 0.88, 1.00) and the pelvic ring (95%CI: 0.94, 1.00). Specificity was poor to moderate in all regions of interest. Positive likelihood ratios (LR+) were of poor value, ranging from 1.48 (95%CI: 1.09, 2.02) in the thoracic spine to 1.08 (95%CI: 1.03, 1.13) in the pelvic ring. Negative likelihood ratios (LR-) were of moderate to good value, ranging from 0.07 in the rib cage (95%CI: 0.01, 0.50) to 0.46 in the thoracic spine (95%CI: 0.20, 1.04). Area under the curve (AUC) values ranged from 0.51 (95%CI: 0.48–0.54) for the lumbar spine to 0.62 (95%CI: 0.59–0.65) for the rib cage.

**Conclusion:**

The presence of physician-documented complaints pertaining to the trunk skeleton have a relevant probability of triggering imaging studies in older persons with LEF in the ED. Nevertheless, these complaints do not safely enable discrimination between individuals with and without fractures and are of limited value modifying the subsequent diagnostic imaging process.

**Supplementary Information:**

The online version contains supplementary material available at 10.1186/s12873-026-01644-2.

## Introduction

Low-energy falls (LEF) are among the most frequent reasons for emergency department (ED) presentation in older adults and are associated with substantial morbidity and mortality [1–5]. In this population, even seemingly minor trauma mechanisms may result in clinically relevant injuries [6, 7].

In the ED, diagnostic work-up of older adults after LEF is initially guided by symptoms, pain localization, and physical examination findings. However, the reliability of such complaints is uncertain in routine emergency care. Older adults frequently present with factors that may complicate symptom assessment, including cognitive impairment, competing acute medical conditions [1, 8] or altered pain perception [9]. Low-energy falls are often underestimated as a significant trauma mechanism, which may further reduce clinical suspicion for relevant skeletal injuries [8, 10–13].

In daily practice, most older adults with LEF present without prior trauma-team activation, either as walk-in patients or via emergency medical services (EMS) [14]. Clinical assessment and imaging decisions are usually made by the treating ED physician based on the initial clinical impression. This approach assumes that patient complaints and documented clinical findings meaningfully modify the pretest probability of fractures. Previous work in the same cohort demonstrated that the prevalence of sustaining a fracture of the truncal skeleton after LEF is approximately 20% [15]. Yet, for several injury regions, especially the thoracolumbar spine, rib cage, and pelvic ring, the diagnostic value of history-taking and physical examination remains insufficiently defined in older patients after LEF [15–23]. Current clinical practice guidelines on imaging of the vertebral column after trauma [24, 25] have been validated for younger patients and high-energy trauma mechanisms.

The aim of the present study was therefore to evaluate the diagnostic value of patient complaints as documented by the treating physician in the ED, specifically in relation to the ordering of radiological imaging and the prediction of fractures of the spine, chest, and pelvic ring in older adults after LEF.

## Methods

### Study design and Setting

This is a secondary analysis of a bicentric, binational retrospective study carried out in two university tertiary care hospitals in Switzerland (University Hospital Basel) and Germany (University Hospital of Ludwig-Maximilians-University Munich) using electronic health record (EHR) data. Parts of the methods used in this study have been previously described [15]. The study is in accordance with the declaration of Helsinki and was conducted using STROBE guidelines. Ethics approval was obtained from local ethics committees (EKNZ 2017 − 01078, EK LMU 17–217).

### Study population

This study represents a distinct secondary analysis of a previously established cohort [15], addressing a different research question and outcome measure. Shortly, the study population included individuals ≥ 65 years of age, presenting to one of the two EDs from 1 January 2016 to 31 December 2016 after a LEF and receiving a CT examination of at least one body region within 48 h of the index visit. All individuals sustained a documented LEF as previously described [15]. Exclusion criteria were: initial presentation via resuscitation bay or following prior trauma team activation, referral from general practitioners or other hospitals with preceding imaging, presentation via fast-track process, delayed presentation (≥ 8 days after the fall), re-presentation due to the same incident, CT examinations of the peripheral extremities or cerebral CT only or radiography only.

### Data collection

In addition to the previously reported baseline demographics, radiological examinations, and injury diagnosis [15], EHR were reviewed for physician-documented complaints and manually extracted from patients’ history and clinical findings. Up to three main complaints per patients were distinguished as follows: no complaint, unknown (no complaint documented), all over body pain, signs of mild concussion (headache, vertigo, amnesia, nausea, vomiting), pain cervical spine, pain thoracic spine, pain lumbar spine, pain pelvic ring, pain extremities, pain rib cage/thorax, sensorimotor disturbances (weakness of limbs, hypo-/paresthesia, cranial nerve disturbances), dyspnea, other (not included in the aforementioned) and more than three complaints. Chart abstraction for all parameters was performed independently by two independent trained observers (AL, RW, IA, NM, VP) in every case. Discrepancies were resolved by a third observer (AL or VP). Screening and chart review abstraction were conducted in accordance with the recommendations for medical chart review [26, 27], which were fulfilled for 11 of 12 guidelines (abstractors were not blinded to the hypothesis). Double data entry was performed in a Microsoft Access 2010/2016 database (Microsoft, Redmond, Washington, USA).

### Outcome measures

The presence of physician-documented complaints and prevalence of fractures of the vertebral column, rib cage and pelvic ring on CT or XR were determined. Secondary measures of diagnostic accuracy of physician-documented complaints to predict fractures were calculated with XR- and/or CT-detected fractures as reference standard, using cross-tabulation (sensitivity and specificity, positive (PPV) and negative predictive values (NPV), positive (LR^+^) and negative likelihood-ratios (LR^-^) [28] and area under the curve (AUC).

### Analyses

For descriptive statistics, medians, and interquartile ranges (IQR) were used for continuous data and frequencies and proportion for counts. For comparisons in categorical and binary values we used Chi^2^ and Fisher test when the expected number values were low and t- test for continuous variables. For diagnostic test performance using the pain in specific region as test and fracture in the same region as the reference standard, we calculated sensitivity, specificity, positive and negative predictive value, and positive and negative likelihood ratios with corresponding 95% confidence intervals (95% CI). Additionally, we calculated an area under the curve (AUC) performance using an univariable logistic regression with 20% jackknife cross validation (repeated 50 times). Confidence intervals were calculated using the DeLong method. Univariable logistic regressions were calculated to assess the association of documented pain in a specific region and a fracture in the same region and compared to multivariable logistic regression including pain in other regions and the interaction of pain in the region of interest and pain in other regions. We calculated the probability of implementation of imaging using odds from univariable logistic regression using pain in the specific region as independent variable and of an emergency imaging of the same region as dependent variable. A p-value < 0.05 was considered significant. Statistical analyses were performed using R version 4.5.1 (The R Foundation for Statistical Computing, Austria) and RStudio version 2025.05.1 + 513 (Posit Software, PBC, Boston, U.S.A).

## Results

**Fig. 1:**
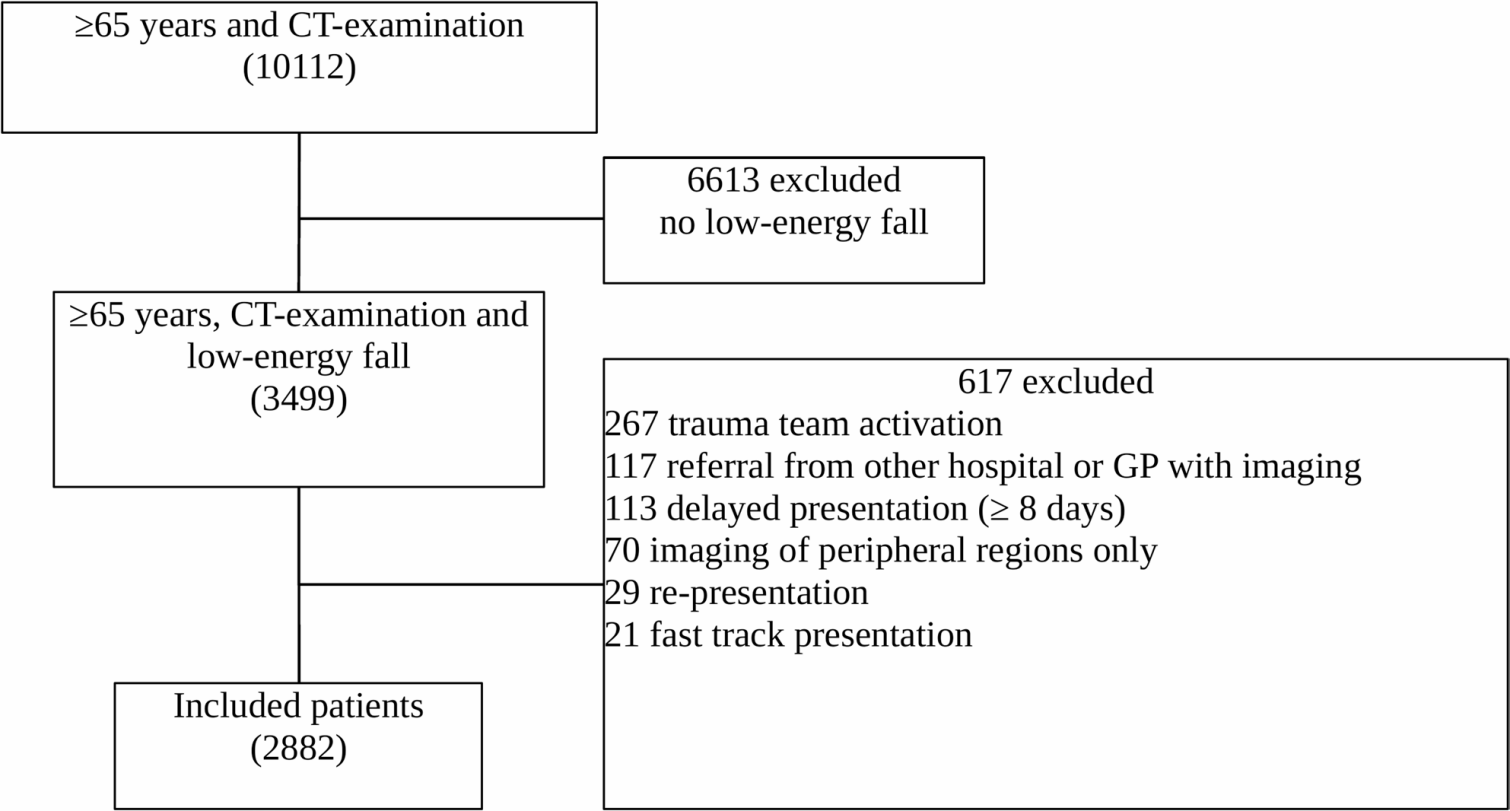
Inclusion and exclusion flow diagram of patient selection between 1 January 2016 and 31 December 2016 in Basel and Munich, receiving computed tomography (CT) examinations of the head, spine, chest, pelvic ring, or proximal long bones during emergency department (ED) presentation or within 48 h. GP: general practitioner; XR: plain radiography

We identified 10,112 cases who presented to the EDs of the two study centers between January 2016 and December 2016 and received CT examinations of any body region. Finally, 2882 cases were included in both centers (Fig. 1) with a median age of 82 years (IQR 76–88), of which 1849 (64.2%) were female. Median injury severity score (ISS) was 3 (IQR 1–5) (Table 1). Baseline characteristics and frequencies and distributions of specific documented complaints are shown in Table 1. The majority of patients (*n* = 1635) received imaging of the cervical spine, 394 received imaging of the thoracic spine, 402 of the lumbar spine, 965 of the thorax, and 665 of the pelvis (Table 2).

**Table 1:** Baseline characteristics

Characteristic	Total *n* = 2882
Female, n (%)	1849 (64.2%)
Age, median [IQR]	82.0 [76.0;88.0]
65–74 years, n (%)	619 (21.5)
75–84 years, n (%)	1151 (39.9)
> 84 years, n (%)	1112 (38.6)
ESI category, n (%)	
1	22 (0.8)
2	282 (9.8)
3	2067 (71.7)
4	496 (17.2)
5	9 (0.3)
Missing	6 (0.2)
Trauma mechanism, n (%)	
Fall from standing, n (%)	2485 (86.2)
Fall from low furniture, n (%)	285 (9.9)
Fall < 1 m, n (%)	112 (3.9)
No physical complaint, n (%)	533 (18.5)
Unknown complaint, n (%)	651 (22.6)
More than three complaints, n (%)	9 (0.3)
Unspecific body pain, n (%)	32 (1.1)
Symptoms of concussion, n (%)	652 (22.6)
Pain cervical spine, n (%)	104 (3.6)
Pain thoracic spine, n (%)	68 (2.4)
Pain lumbar spine, n (%)	155 (5.4)
Pain pelvis, n (%)	340 (11.8)
Pain extremities, n (%)	489 (17.0)
Pain rib cage thorax, n (%)	142 (4.9)
Sensory-motor deficit, n (%)	98 (3.4)
Dyspnea, n (%)	20 (0.7)
Other, n (%)	122 (4.2)
Supraclavicular injury signs	1415 (49.0)
ISS, median [IQR]	3.00 [1.00;5.00]
ISS ≥16, n (%)	86 (3.0)
ISS = 0, n (%)	382 (13.3)

ESI: emergency severity index; ISS: injury severity score

**Table 2:** Summary of probability (95% CI) for imaging and association (odds ratio, 95% CI) for imaging in a specific region if pain is documented

Region	imaging series (*n*)	Patients with pain (*n*)	Probability	OR	*p*
CS	1635 (CT: 1622, XR: 37, CT&XR: 24)	104	0.99 (0.95, 1.00)	63.06 (12.93, 1139.2)	< 0.001***
TS	394 (CT: 309, XR: 128, CT&XR: 43)	68	0.82 (0.71, 0.91)	5.86 (2.84, 12.88)	< 0.001***
LS	402 (CT: 268, XR: 217, CT&XR: 83)	155	0.87 (0.80, 0.91)	13.05 (6.69, 26.51)	< 0.001***
Chest	965 (CT: 266, XR: 775, CT&XR: 76)	142	0.91 (0.85, 0.95)	10.38 (5.25, 21.78)	< 0.001***
PR	665 (CT: 348, XR: 513, CT&XR: 196)	340	0.93 (0.90, 0.95)	32.6 (17.26, 64.2)	< 0.001***

95%CI: 95% confidence interval; CS: cervical spine; CT: computed tomography; LS: lumbar spine; OR: odds ratio; PR: pelvic ring; TS: thoracic spine; XR: X-ray. *p* < 0.001***

### Imaging probability

Generally, the probability of receiving an XR- or CT-image of the region after documenting pain in the same region was high (Table 2): In patients with documented pain in the cervical spine, the probability is 0.99 (95% CI: 0.95, 1.00), in patients with documented pain in the thoracic spine the probability is 0.82 (95% CI: 0.71, 0.91), in patients with documented pain in the lumbar spine the probability is 0.87 (95% CI: 0.80, 0.91), in patients with documented pain of the thorax the probability is 0.91 (95% CI: 0.85, 0.95) and in patients with documented pain of the pelvis the probability of imaging is 0.93 (95% CI: 0.9, 0.95). In all observed body regions of the body trunk there were significant associations with XR- or CT-imaging when pain was documented (Table 2).

### Diagnostic test performance

**Fig. 2:**
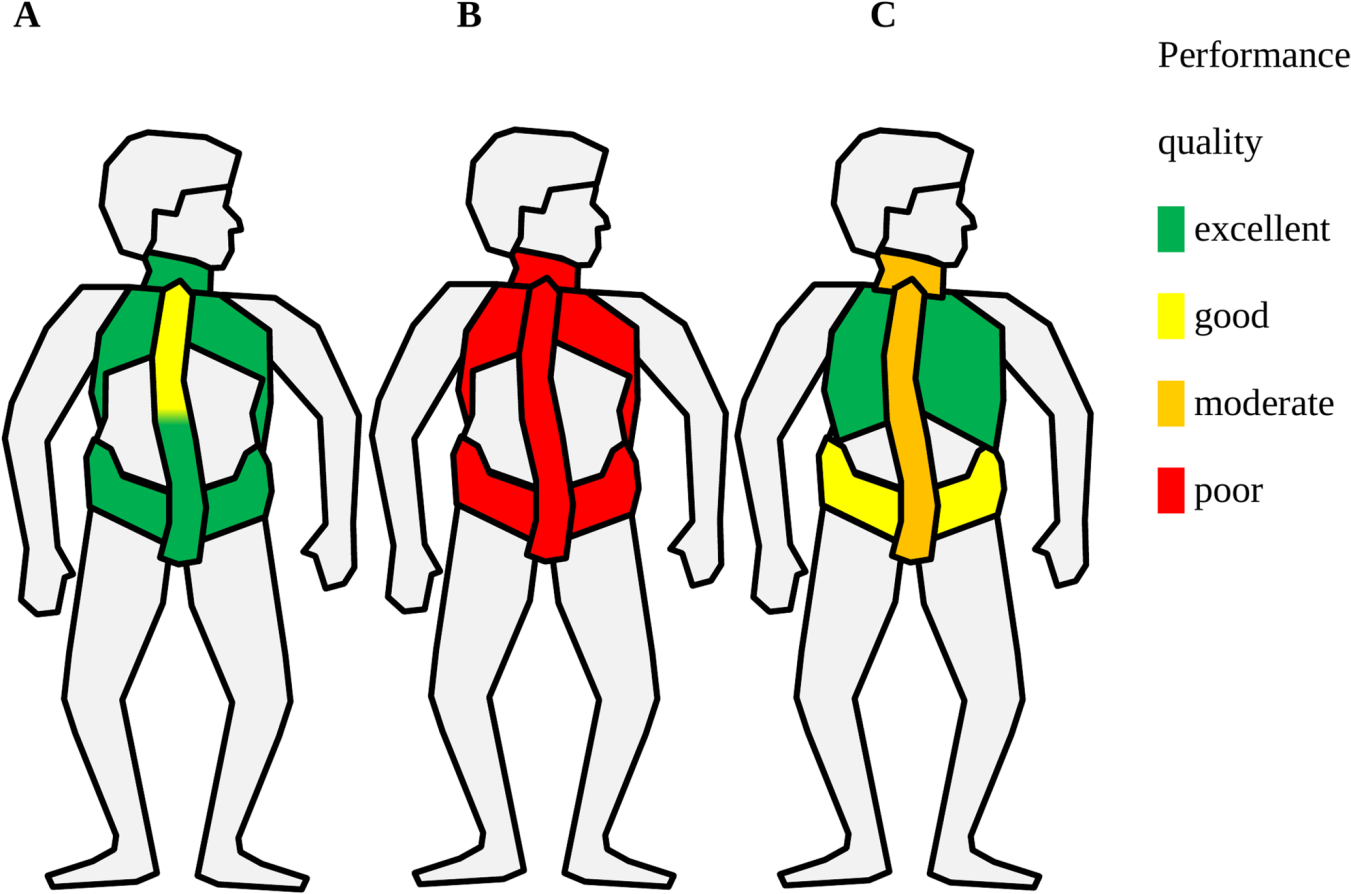
Performance quality of specified documented complaints (pain cervical spine, pain thoracic spine, pain lumbar spine, pain chest/thorax, pain pelvis) to predict fractures of the determined body regions (cervical spine, thoracic spine, lumbar spine, thorax/rib cage, pelvic ring) revealed by applied imaging modalities. Measures include (**A**) sensitivity, (**B**) positive likelihood ratio (LR+) and (**C**) negative likelihood ratio (LR-). Performance cut-offs are determined as: excellent: 80–100% (sensitivity), > 10 (LR+), < 0.1 (LR-); good: 60–80% (sensitivity), 5–10 (LR+), 0.1–0.2 (LR-); moderate: 40–60% (sensitivity), 2–5 (LR+), 0.2–0.5 (LR-); poor: 0–40% (sensitivity), 1–2 (LR+), 0.5–1 (LR-) [29, 30]. Body illustration modified from [44] and kindly provided by R. Lefering. n.a.: not applicable

We analyzed the diagnostic test performances using pain in a region as a test to diagnose a fracture in the same region using either XR or CT as reference standard (Table 3). Overall, the apparent fracture prevalence ranged from 0.59 (95% CI: 0.48, 0.69) for fractures of the thoracic spine to 0.94 (95% CI: 0.91, 0.96) for fractures of the pelvic ring. The estimated true prevalence ranged from 0.14 (95% CI: 0.09, 0.20) for fractures of the cervical spine to 0.36 (95% CI: 0.30, 0.41) (Table 2). The sensitivity of documented pain in a specific region for a fracture in this region was moderate in the thoracic spine (0.78, 95% CI: 0.56, 0.93), and high in the other regions: cervical spine 0.90 (95% CI: 0.70, 0.99), lumbar spine 0.92 (95% CI: 0.81, 0.98), rib cage 0.98 (95% CI: 0.88, 1.00) and pelvic ring 0.98 (95% CI: 0.94, 1.00). Specificity of documented pain ranged from 0.09 (pelvic ring) to 0.47 (thoracic spine). Cross validation AUC values ranged from 0.51 (lumbar spine) to 0.62 (rib cage), indicating that detection of a fracture in a specific region is almost as likely when pain is documented as if pain is not documented (Table 3). Figure 2 illustrates performance cut-offs (from poor to excellent) [29, 30] for sensitivity, LR + and LR- of the specified documented pain in cervical spine, thoracic spine, lumbar spine, chest/thorax and pelvic ring to predict fractures of the specified body regions (cervical spine, thoracic spine, lumbar spine, thorax/rib cage, pelvic ring).

**Table 3:** Summary of diagnostic performance of documented pain in a specific region for a fracture in the same region in XR or CT

Region	Apparent Prevalence	True Prevalence	Sensitivity (%)	Specificity (%)	PPV (%)	NPV (%)	LR+	LR-	AUC	Cross validation AUC
CS	0.68 (0.60, 0.75)	0.14 (0.09, 0.20)	0.90 (0.70, 0.99)	0.36 (0.28, 0.45)	0.18 (0.11, 0.27)	0.96 (0.86 1.00)	1.41 (1.17, 1.70)	0.27 (0.07, 1.01)	0.63 (0.55, 0.71)	0.55 (0.51, 0.59)
TS	0.59 (0.48, 0.69)	0.24 (0.16, 0.34)	0.78 (0.56, 0.93)	0.47 (0.35, 0.59)	0.32 (0.20, 0.46)	0.87 (0.73, 0.96)	1.48 (1.09, 2.02)	0.46 (0.20, 1.04)	0.63 (0.52, 0.73)	0.55 (0.50, 0.59)
LS	0.86 (0.79, 0.91)	0.33 (0.26, 0.41)	0.92 (0.81, 0.98)	0.17 (0.11, 0.26)	0.36 (0.28, 0.45)	0.82 (0.60, 0.95)	1.12 (0.99, 1.26)	0.44 (0.16, 1.25)	0.55 (0.49, 0.60)	0.51 (0.48, 0.54)
Thorax	0.75 (0.68, 0.81)	0.25 (0.19, 0.32)	0.98 (0.88, 1.00)	0.33 (0.25, 0.41)	0.33 (0.25, 0.41)	0.98 (0.88, 1.00)	1.45 (1.27, 1.65)	0.07 (0.01, 0.50)	0.65 (0.60, 0.70)	0.62 (0.59, 0.65)
PR	0.94 (0.91, 0.96)	0.36 (0.30, 0.41)	0.98 (0.94, 1.00)	0.09 (0.05, 0.13)	0.37 (0.32, 0.43)	0.90 (0.70, 0.99)	1.08 (1.03, 1.13)	0.19 (0.05, 0.80)	0.53 (0.51, 0.56)	0.52 (0.50, 0.54)

95%CI: 95% confidence interval; AUC: area under the curve; CS: cervical spine; CT: computed tomography; LS: lumbar spine; OR: odds ratio; PR: pelvic ring; SI: supraclavicular injury; TS: thoracic spine; XR: X-ray. p 0.05–0.1#, *p* < 0.05*, *p* < 0.001**

### Prediction and interaction terms

Next, we evaluated if pain in adjacent regions is predictive for fractures in the region of interest (Table 4). Presence of pain in the lumbar spine was significantly associated with a reduced risk for a fracture in the cervical spine (OR: 0.08, 95%CI: 0.00, 0.80, p: 0.03). Supraclavicular injury signs were not significantly associated with fractures of the cervical spine (OR: 2.28, 95%CI: 0.36, 25.3, p: 0.4). There was no significant association of pain in the lumbar spine with fractures of the thoracic spine (OR: 5.60, 95%CI: 0.84, 66.46, p: 0.08), or pain in the thoracic spine with fractures of the lumbar spine (OR: 3.15, 95%CI: 0.81, 12.44, p: 0.1), or pain of the rib cage and thorax with fractures of the rib cage (OR: 5.87, 95%CI: 0.86, 75.77, p: 0.07). All models demonstrate that the basic probability for a fracture in the determined region is very low in the absence of pain (see intercept values in Table 4). Supplementary Table S1 summarizes interaction analysis. Overall, the total number of patients who exhibited combinations of the respective predictors where interactions could be calculated, is small. None of the interaction models show a significant prediction of fractures when pain is observed in the defined region together with pain in an adjacent region.

**Table 4:** Summary of prediction of fractures in specific regions with documented pain in the determined or adjacent regions. Odds ratio with 95% CI

Region	Patients with fractures (*n*)	Pain CS	*p*	Pain TS	*p*	Pain LS	*p*	Pain thorax	*p*	Pain PR	*p*	SI	*p*	Interception	LR test (*p*)
CS	46	2.52 (0.43, 17.61)	0.30	0.23 (0.0, 2.58)	0.27	0.08 (0.00, 0.80)	0.03^*^	n.a.	-	n.a.	-	2.28 (0.36, 25.3)	0.4	0.07 (0.01, 0.39)^**^	0.17
TS	73	2.77 (0.59, 14.55)	0.19	2.13 (0.40, 12.33)	0.37	5.60 (0.84, 66.46)	0.08^#^	1.33 (0.12, 10.35)	0.79	n.a.	-	0.23 0.03, 1.71)	0.14	0.13 (0.01, 1.01)^#^	0.38
LS	80	0.88 (0.14, 4.26)	0.87	3.15 (0.81, 12.44)	0.10#	0.93 (0.24, 4.02)	0.92	n.a.	-	1.32 (0.31, 5.71)	0.70	n.a.	-	0.15 (0.03, 0.58)^**^	0.51
Thorax	95	0.23 (0.00, 2.39)	0.26	1.01 (0.09, 6.81)	0.99	0.82 (0.07, 7.81)	0.86	5.87 (0.86, 75.77)	0.07^#^	n.a.	-	n.a.	-	0.07 (0.01, 0.40)^**^	0.14
PR	153	n.a.	-	1.90 (0.28, 10.89)	0.48	0.98 (0.22, 4.39)	0.48	n.a.	-	1.99 (0.48, 11,39)	0.36	n.a.	-	0.12 (0.02, 0.52)^**^	0.87

95% CI: 95% confidence interval; CS: cervical spine; n.a.: not applicable; LR: likelihood ratio; LS: lumbar spine; OR: odds ratio; PR: pelvic ring; SI: supraclavicular injury; TS: thoracic spine. p 0.05–0.1#, *p* < 0.05*, *p* < 0.001**

## Discussion

The main finding of this retrospective bi-centric study is the association of emergency physician-documented complaints with the indication of emergency imaging of the vertebral column, thorax/rib cage and pelvic ring following a LEF in the older individual. In this cohort, apparent fracture prevalence, derived from patients who underwent imaging, ranged from 0.59 (thoracic spine) to 0.94 (pelvic ring). Estimated true prevalence were lower, as asymptomatic patients or patients without documented complaints were less likely to receive imaging. With this basic condition, the rating of NPV to rule out fractures is not reliable. Based on our own previously published data, we estimate the overall pretest-probability for fractures of the torse following a LEF at 20% [15]. The calculated LR + for the specified regions do not provide strong diagnostic evidence to increase post-test probability for a fracture, with a marginal increase of probability from 20% to 27% at best. With the calculated LR- of the recent data, the post-test probability was reduced to clinical more meaningful 1.7% (thorax/rib cage) and 4.5% (pelvic ring). Logistic regression and interaction models support the findings that none of the documented complaints significantly predicts fractures in the determined regions, even if pain in an adjacent region is considered. In general, the basic probability for detection of a fracture by emergency imaging is low in the absence of documented pain. However, cross validation AUC analysis revealed that the initial patient assessment and physical examination of the patients, does not allow to reliably discriminate between older individuals with fractures from those without fractures following a LEF.

The most frequent complaint documented by ED physicians in older patients with LEF were signs of mild traumatic brain injury. Injury signs above the clavicle, like bruises or lacerations, were documented in nearly half of all patients. The overall prevalence of traumatic intracranial hemorrhage in this cohort was 6.9%, as we previously published [31], with supraclavicular injury signs, but not headache or reduced GCS, as stronger predictor of traumatic intracranial hemorrhage [31–35].

Interestingly, in 18.5% of cases, ED physicians documented that the patients presented without any complaints after LEF. Of these, 6.8% were moderately to severely injured, according to an injury severity score of 9 or more. Only 11.4% of patients presenting without complaints sustained a non-injurious fall. This might question the reliability of the initial assessment of older patients with LEF and may be a sign of a systematic underestimation of the older patient with low-energy trauma [10–13]. From a patient’s perspective, this might include a reduced pain perception [9], masking injuries [36], severe medical or neurological conditions and preexisting cognitive or sensory deficits that confound history taking and clinical examination [37]. From the caregivers and physicians’ perspective, the erroneous assumption that a minor trauma mechanism is associated with no or minor injuries, might result in disregarding or ignoring even nonspecific or variable complaints in a relevant proportion of patients.

Evidence on the reliability of history taking and physical examination in older adults with LEF for detecting injuries is limited and inconsistent. A large trauma registry analysis reported a sensitivity of 0.69 for injury detection by physical exam compared with pan-scan CT [23]. In contrast, an all-age trauma registry evaluation found that only a positive physical exam, rather than age, was associated with increased odds of abnormal torso CT [38]. More broadly, clinical examination appears to have limited ability to identify even life- or limb-threatening injuries, even when performed by the most experienced trauma clinicians [29]. Our cohort consisted solely of older individuals not treated in the trauma bay after LEF, representing the majority of older LEF presentations [14]. For this large cohort of older adults, presenting without trauma-team activation to the ED following a LEF, our findings now further support the assumption, that physical examination and pain assessment only provide limited accuracy for fracture detection in older patients with blunt, low-energy transfer trauma.

Registry data demonstrate that older trauma patients are more likely to be assessed and examined by the most junior and inexperienced doctors in the ED [6]. This applies to many EDs, and, therefore, might inherit a relevant interobserver variability in patient assessment, examination and documentation of the findings. This constitutes a further possible explanation for a relatively inaccurate or superficial medical history and physical examination in our study cohort.

In cases with physician-documented complaints, our data showed a strong association between reported pain in specific skeletal regions and the use of XR- or CT-imaging. Pain of the cervical spine pain was recorded only in a minority of our patients. However, local standards based on the Canadian C-Spine Rule and NEXUS criteria [39, 40] recommend a low threshold for CT-imaging in individuals of 65 years of age and older, regardless of pain. This resulted in an extensive use of cervical spine CT in these patients, resulting in a very small proportion of symptom negative and imaging negative patients. Nevertheless, the presence or absence of documented cervical pain did not discriminate between injured and uninjured patients. Furthermore, likelihood ratios both did not provide strong diagnostic evidence to support physician-documented cervical spine pain for rule-in or rule-out fall-related injuries of the cervical spine. In line with this, prior smaller studies similarly report limited diagnostic accuracy of cervical spine tenderness in older adults [41]. While facial or frontal trauma combined with midline tenderness has been linked to cervical spine injury [42, 43], we found no significant association between supraclavicular injury signs and cervical spine fractures. However, given recent evidence that supraclavicular injury signs are associated with intracranial hemorrhage in older LEF patients [31, 32, 34], CT-imaging of the head and cervical spine may be justified when these signs are present, potentially simplifying imaging decisions.

Imaging studies diagnosed fractures of the thoracic and lumbar spine in one out of four examinations of these regions. However, pain in the thoracic and lumbar spine was not predictive for fractures, but the possibility of a fracture in these regions was very low when pain was absent. Furthermore, our data showed that pain in the thoracic spine could be associated with fractures of the lumbar spine and vice versa. This might possibly reflect an unprecise pain localization of the patient or the examiner. A previous meta-analysis demonstrated a low diagnostic accuracy of injury mechanism and physical examination to rule-in or rule-out fractures of the thoracolumbar spine after blunt injury in adult patients [22]. This study also favored CT-imaging over plain radiography for accurate fracture detection [22]. Taking into account that one out of three fractures would have been missed in plain radiography [15], our data emphasizes the use of immediate CT-examination of these two spine regions as soon as an injury is suspected or clinical symptoms are present, possibly also across both regions by default.

### Limitations

Our analysis should be interpreted in light of both methodological strengths and important limitations. A major strength is the inclusion of a large consecutive cohort from two representative urban tertiary care centers in Europe, combined with a structured chart abstraction process for key eligibility criteria and study variables. This provides a clinically relevant dataset that reflects real-life routine emergency care for older adults after LEF, exclusively presenting to the ED without prior trauma team activation. However, several limitations must be considered. First, the retrospective nature of the study restricts the degree to which causal or clinically actionable conclusions can be drawn. Second, the cohort assembly and diagnostic work-up may have introduced selection bias that could have influenced the observed fracture prevalence. In particular, patients without symptoms in a given body region were less likely to undergo imaging of that region, which may have biased the assessment of diagnostic performance, resulting in a verification bias. Third, despite the high quality of the data extraction from the EHR in parallel by two independent abstractors, the present study relied on physician-documented complaints rather than directly recorded patient-reported symptoms. This distinction is important, because clinicians may perceive LEF as minor trauma and may therefore incompletely recognize, asses or document complaints that would later prove clinically relevant. Such an expectation bias may contribute to under-recognition of patient symptoms and may reduce the apparent association between reported complaints and confirmed injuries. Given the varying levels of experience among the treating physicians, we must further assume that there is significant inter-observer variability, which leads to bias in both the clinical assessment of patients and the documentation, and consequently to an under-recording bias. Finally, because of the retrospective design, the direct clinical implications of these findings remain uncertain. Nevertheless, the study highlights relevant limitations and weaknesses in the initial emergency assessment of older adults after LEF. The findings of this study lend support to the argument that patient complaints and clinical findings ought to be documented in a more structured manner, which could then serve as a basis for future more patient-centered approaches and decision-support algorithms.

## Conclusions

Pain in specific skeletal regions of the trunk in older patients after LEF is associated with the execution of emergency imaging by XR and/or CT. However, documented complaints in these regions do not enable reliable discrimination between individuals with and without fractures during the initial assessment in the ED. This suggests a low threshold for ancillary imaging and supports recommendations from previous studies. Prospective studies, focusing on patient-reported complaints, rather than physician-documented complaints, including systematic and structured reporting systems are required to shed more light on the reliability of the initial assessment of older patients with LEF and their potential injuries and to define patient-centred management and resource-oriented pathways.

## Supplementary Information

Below is the link to the electronic supplementary material.

Supplementary Material 1

## Data Availability

The datasets used and/or analyzed during the current study are available from the corresponding author on reasonable request.

## Abbreviations

AUC: Area under the curve
CI: Confidence interval
CT: Computed tomography
ED: Emergency department
HER: Electronic health record
IQR: Interquartile range
LR+: Positive likelihood ratio
LR-: Negative likelihood ratio
NPV: Negative predictive value
LEF: Low energy fall
OR: Odds ratio
PPV: Positive predictive value
XR: Plain radiography
