# Supplementary material for: Pain has limited predictive value for fractures following falls in older people: insight from a retrospective study
Source: BMC Emerg Med. 2026 Jun 16;26:176. doi: 10.1186/s12873-026-01644-2 (PMC13277108; doi:10.1186/s12873-026-01644-2)
Supplement: Supplementary file 1 — Supplementary Material 1 [file 12873_2026_1644_MOESM1_ESM.docx]

Supplementary Table S1. Summary of interaction analysis of documented pain in the determined and adjacent regions and fractures in the determined regions. Odds ratio with 95% CI.

| Region | sample size^1^ (n) | Pain CS | p | Pain TS | p | Pain LS | p | Pain thorax | p | Pain PR | p | SI | p | LR test | p |
| --- | --- | --- | --- | --- | --- | --- | --- | --- | --- | --- | --- | --- | --- | --- | --- |
| CS | 80 | **4.67 (0.21, 720.9)** | **0.33** | 0.28 (0.001, 69.7) | 0.58 | 0.71 (0.003, 168.0) | 0.88 | n.a. | - | n.a. | - | 0.43 (0.002, 16.8) | 0.66 | 5.48 | 0.6 |
| TS | 52 | 0.24 (0.006, 6.2) | 0.39 | **11.4 (0.2, 3373)** | **0.24** | 0.08 (0.0001, 9.4) | 0.32 | 3.4 (0.05, 1008) | 0.58 | n.a. | - | 1.71 0.03, 107) | 0.78 | 7.36 | 0.6 |
| LS | 61 | 14.8 (0.5, 2850) | 0.12 | 0.46 (0.02, 7.24) | 0.59 | **0.37 (0.03, 5.83)** | **0.45** | n.a. | - | 4.50 (0.26, 104) | 0.30 | n.a. | - | 6.40 | 0.49 |
| Thorax | 65 | 0.35 (0.001, 90) | 0.66 | 1.99 (0.04, 435) | 0.74 | 0.18 (0.0004, 11) | 0.43 | **10.25 (0.91, 1411,8)** | **0.06^#^** | n.a. | - | n.a. | - | 7.36 | 0.39 |
| PR | 57 | n.a. | - | 2.31 (0.06, 464) | 0.67 | 9.93 (0.41, 1608) | 0.16 | n.a. | - | **0.51 (0.07, 3.91)** | **0.50** | n.a. | - | 3.99 | 0.55 |

95% CI: 95% confidence interval; CS: cervical spine; n.a.: not applicable; LR: likelihood ratio; LS: lumbar spine; OR: odds ratio; PR: pelvic ring; SI: supraclavicular injury; TS: thoracic spine. p 0.05-0.1#, p<0.05*, p<0.001**. ^1^Total number of cases who exhibited combination of the respective predictors where interactions could be calculated.
